# Threat from Emerging Vectorborne Viruses

**DOI:** 10.3201/eid2205.160284

**Published:** 2016-05

**Authors:** Ronald Rosenberg

**Affiliations:** Centers for Disease Control and Prevention, Fort Collins, Colorado, USA

**Keywords:** threat, viruses, Zika virus, dengue virus, chikungunya virus, arbovirus, vector-borne viruses, vector-borne infections, vector, emerging pathogen, mosquitoes

The earliest members of genus *Homo* were surely bedeviled by blood-feeding arthropods, some of which doubtless carried zoonotic pathogens. However, the phenomenon of vectorborne human epidemic disease began only after humans began building settlements 15,000 years ago (*1*). Settlements offered pathogens not only host density but also opportunities for their vertebrate reservoirs and arthropod vectors to cohabit with us. Epidemic *Yersinia pestis* (the Medieval Black Death) was only possible because black rats (*Rattus rattus*), the host of the vector flea, had become extraordinarily successful at living off human garbage and nesting in our buildings.

Two of the most important malaria vectors in the world exploit human activity to proliferate. Immature forms of *Anopheles gambiae* mosquitoes in Africa and *An. dirus* mosquitoes in Southeast Asia thrive in the small puddles (water-filled footprints, tire ruts, borrow pits, and drainage gullies) created around villages. Best adapted of all are *Aedes aegypti* mosquitoes, the cosmopolitan vector of epidemic yellow fever, dengue, chikungunya, and Zika viruses. Their ecologic niche is nearly ours. These mosquitoes lay eggs in artifacts: water storage jars, roof gutters, flower pots, dog dishes, even upturned bottle caps. Their cognate species, *Ae. albopictus*, is only slightly less versatile, having an attraction to discarded tires. Evolution of blood-feeding arthropods to our changing environment and evolution of some zoonoses to exploit this advantage are major links in the emergence of obscure pathogens into epidemic threats and is a timely subject for this issue of Emerging Infectious Diseases.

Persistence of human yellow fever, the seeming inexorable expansion of dengue, and the surprising, explosive spread and severity of first chikungunya virus and now Zika virus bear testament to the threat posed by habituated *Aedes* species. Since its arrival in the Western Hemisphere ≈1 year ago, Zika virus, which had previously been associated with a clinically mild and inconsequential illness, is now increasingly suspected of being the cause of an alarming epidemic of neurologic birth defects and Guillain-Barré syndrome in tropical regions. Zika virus, the subject of several articles in this issue, reminds us of some of the impediments to responding to emerging vectorborne pathogens.

First, Zika virus belongs to the most prevalent class of emerging pathogens, the zoonotic single-stranded RNA viruses, which have mutation rates as high as 1 base/10^4^ bases each replication. The chikungunya pandemic that began 10 years ago was fueled in part by a single, nonsynonymous base change that enabled that alphavirus to replicate more efficiently in *Ae. albopictus* mosquitoes (*2*).

Second, conditions enabling transition from vectorborne animal-to-animal transmission to arthropod-mediated human-to-human transmission are poorly understood. Like dengue virus, another flavivirus, Zika virus was likely originally a pathogen of subhuman primates. Between its discovery in a sentinel macaque in Uganda in 1947 and the first recorded epidemic 60 years later in Yap, Federated States of Micronesia, only 14 human cases had been reported, all from Africa and Asia (*3*).

Third, the pathogenicity and transmission dynamics of vectorborne zoonotic pathogens are much more complex than those of directly communicable pathogens. It is not yet known if Zika virus will find sustaining, nonhuman hosts in the Western Hemisphere, as has yellow fever virus, or how wide the range of vector species will be. Pathogenicity and transmission dynamics will be factors in determining where Zika virus will become endemic and what will be the most suitable methods of control.

Fourth, accurate diagnosis is key to surveillance and response. It might seem as if Zika virus sprang from nowhere, but almost certainly it must have been infecting many more humans in Africa and Asia than we had been aware. Our ability to serologically diagnose infections with emerging arboviruses is often compromised by close antigenic relationships within virus families. Zika, dengue, West Nile, and yellow fever viruses can co-circulate, not only among themselves, but possibly with unidentified or poorly characterized flaviviruses. The limitations of current diagnostics are a primary reason why the association between Zika virus and birth defects remained speculative so long.

Fifth, vector control is a force multiplier that can reduce the risk from many viruses that would require the development of individual vaccines. However, insecticide resistance and application problems greatly impede effective implementation.

The best defense is preventing a problem from growing into a threat. Fewer than 20 of the 86 known pathogenic arboviruses can be considered major causes of human disease, and 3 of these, West Nile, chikungunya, and Zika viruses, have emerged from relative obscurity within only the past 20 years (*4*). At least another 200 cataloged arboviruses whose relationship to human disease is unknown have been isolated from arthropods or animals.

The discovery of 3 highly pathogenic mosquitoborne viruses in China and the United States during the past 5 years (*5**–**7*) underscores how unrepresentative even that large number might be. It is unrealistic to characterize each of these viruses. Besides needing better methods of vector control, we need a strategy for preemptively identifying arboviruses with the potential for emergence and to devote resources to better understand their transmission dynamics, their endemicity, and accurate diagnosis.

## References

[1] Diamond J. Guns, germs and steel. New York: Norton; 1997.

[2] de Lamballerie X, Leroy E, Charrel RN, Ttsetsarkin K, Higgs S, Gould EA. Chikungunya virus adapts to tiger mosquito via evolutionary convergence: a sign of things to come? Virol J. 2008;5:33. 10.1186/1743-422X-5-33 18304328 PMC2266737

[3] Hayes EB. Zika virus outside Africa. Emerg Infect Dis. 2009;15:1347–50. 10.3201/eid1509.090442 19788800 PMC2819875

[4] Rosenberg R, Johansson M, Powers A, Miller B. Search strategy has influenced the rate of discovery of human viruses. Proc Natl Acad Sci U S A. 2013;110:13961–4. 10.1073/pnas.1307243110 23918354 PMC3752202

[5] Yu XJ, Liang MF, Zhang SY, Liu Y, Li JD, Sun YL, . Fever with thrombocytopenia associated with a novel bunyavirus in China. N Engl J Med. 2011;364:1523–32. 21410387 10.1056/NEJMoa1010095 PMC3113718

[6] McMullan LK, Folk SM, Kelly AJ, MacNeil A, Goldsmith CS, Metcalfe MG, . A new phlebovirus associated with severe febrile illness in Missouri. N Engl J Med. 2012;367:834–41 22931317 10.1056/NEJMoa1203378

[7] Kosoy OI, Lambert AJ, Hawkinson DJ, Pastula DM, Goldsmith CS, Hunt DC, . Novel thogotovirus associated with febrile illness and death, United States, 2014. Emerg Infect Dis. 2015;21:760–4. 25899080 10.3201/eid2105.150150 PMC4412252

